# Genetic Variability of *Pestivirus A* (BVDV‐1) Circulating in Cattle From Eastern Turkey

**DOI:** 10.1002/vms3.70127

**Published:** 2025-01-10

**Authors:** Fatima Abounaaja, Ali Riza Babaoglu

**Affiliations:** ^1^ Department of Virology, Institute of Health Sciences Van Yuzuncu Yil University Van Turkey; ^2^ Department of Virology, Faculty of Veterinary Medicine Van Yuzuncu Yil University Van Turkey

**Keywords:** bovine viral diarrhoea virus, phylogenetic analysis, subgenotyping

## Abstract

**Background:**

Bovine viral diarrhoea virus (BVDV) infection, caused by *Pestiviruses A* and *B*, with various clinical findings and causes significant economic losses. This disease is common in Turkey as well as in other countries, especially in European countries.

**Objective:**

This study was designed to determine the genotypes of BVDVs and their variability among cattle in eastern Turkey.

**Methods:**

A total of 110 samples from 85 cattle suspected of BVDV infection were tested using RT‐PCR with primers targeting the *5′UTR*, *autoprotease (N^pro^)* and *E2* gene regions of pestiviruses. Sequence and phylogenetic analyses were performed on the *5′UTR* and *N^pro^* gene regions of these samples.

**Results:**

Analysis of 15 sequences obtained from 13 cattle revealed that *Pestivirus A* (BVDV‐1) was responsible for the infection. In addition, the study identified subgenotypes BVDV‐1*a* (*n* = 5), 1*b* (*n* = 5), 1*d* (*n* = 1), 1*f* (*n* = 1), 1*l* (*n* = 1) and 1*r* (*n* = 2). No evidence of infection with *Pestivirus B* (BVDV‐2), *Pestivirus* D (Border disease virus) or *Pestivirus H* (HoBi‐like virus/BVDV‐3) was found.

**Conclusion:**

The significance of pestiviruses in causing genital and respiratory problems is once again emphasised, underscoring the necessity of including them in herd screening. Identifying BVDV genetic diversity both in Turkey and worldwide is crucial for developing effective protection, control and eradication strategies, particularly for vaccination programs. As a conclusion, the identification of BVDV‐1*a*, 1*b*, 1*d*, 1*f*, 1*l* and 1*r* in the eastern provinces of Turkey points to an increase in BVDV‐1 genetic diversity.

## Graphical abstract

This study investigated the genotypes and variability of bovine viral diarrhoea virus (BVDV) in cattle from eastern Turkey. Using RT‐PCR with primers targeting the *5′UTR*, *autoprotease (N^pro^)* and *E2* genes and sequence and phylogenetic analysis, 15 sequences from cattle were examined. The findings identified *Pestivirus A* (BVDV‐1) with several subgenotypes, including BVDV‐1*a*, 1*b*, 1*d*, 1*f*, 1*l* and 1*r*. The study highlights the importance of screening for BVDV due to its role in causing genital and respiratory issues and emphasises the need for effective control and eradication strategies, including vaccination, to manage BVDV's genetic diversity.

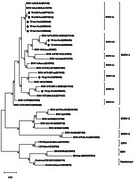

## 1 Introduction

Bovine viral diarrhoea virus (BVDV) is an important infectious agent in cattle populations and has a global distribution, resulting in reduced meat, milk, reproductive performance and substantial economic impacts (Houe 1999). BVDV is classified in the *Pestivirus* genus within the family *Flaviviridae*. Two genotypes of BVDV have been identified worldwide: BVDV‐1 and BVDV‐2. According to the International Committee on Taxonomy of Viruses (ICTV), BVDV‐1 and BVDV‐2 are now referred to as *Pestivirus A* and *Pestivirus B*, respectively (Smith et al. 2017; Simmonds et al. 2017). These were categorised into multiple subgroups, including more than 21 in *Pestivirus A* (designated a through u) and 4 in *Pestivirus B*. Other recognised pestiviruses include *Pestivirus C* (classical swine fever virus), *Pestivirus D* (border disease virus) and *Pestivirus H* (BVDV‐3 or HoBi‐like pestivirus). In addition, an increasing number of pestivirus species have been identified from different species (Becher et al., 2003; Vilcek, Ridpath et al. 2005; Bauermann et al. 2013). Cattle are recognised as the natural hosts for BVDV‐1, BVDV‐2 and BVDV‐3; however, these viruses are also capable of infecting other cloven‐hoofed animals (Evans and Reichel 2021).

The BVDV genome is composed of an RNA that is 12.3 kb in length. The viral genome encodes a single open reading frame, which is flanked by untranslated regions (*UTRs*). The *5′UTR* region is composed of about 360–390 nucleotides, and the *3′UTR* region consists of 200–240 nucleotides (Ridpath 2005). A major obstacle in managing pestivirus infections is the significant genetic diversity of BVD viruses present across the globe (Tautz, Tews, and Meyers 2015). Analysing the *5′UTR*, *N‐terminal protease* (*N^pro^*), and envelope glycoprotein *E2* gene regions allows for a more accurate understanding of the global distribution of the virus through molecular characterisation and genotyping of subgroups. The *5′UTR* gene region, which contains conserved motifs in pestiviruses, is often preferred in both PCR and molecular characterisation studies because it offers more opportunities for comparison with reference strains in GenBank. Numerous studies on BVDV genome variability have concentrated on the *5′ UTR* region due to its relative conservation. However, the *N^pro^* and *E2* gene regions are also used in molecular analyses (Vilcek et al. 1994; Vilcek et al. 2004; Vilcek, Durkovic, et al. 2005; Yeşilbağ, Alpay, and Becher 2017; Oğuzoğlu et al. 2019; Timurkan and Aydın 2019). In addition, the study of BVDV genotypes in specific geographical areas is essential both clinically and biologically due to the antigenic differences among BVD virus subtypes. Consequently, understanding the variability in these gene regions is crucial for developing effective vaccines (Walz et al. 2020).

Pestiviruses are a major contributor to bovine genital tract issues in ruminants, and these infections are reproductive diseases closely linked to herd management practices. BVDVs spread through secretions, vectors and vertical transmission, causing respiratory, intestinal and reproductive issues. Vertical transmission during pregnancy can lead to malformations, abortions or the birth of immune‐tolerant persistently infected (PI) calves. PI animals continuously shed large quantities of the virus throughout their lifetimes, posing a significant risk to healthy animals and complicating disease control efforts (Houe 1999; Lanyon, Hill, and Reichel 2014).

In Turkey, the prevalence and genetic diversity of BVDV vary depending on geographical location and animal imports. This virus is common in Turkey, impacting both dairy and beef herds. Studies on this virus in Turkey have frequently reported the existence of BVDV‐1 and BVDV‐2 subtypes and BVDV‐3 (HoBi‐like pestivirus) was reported for the first time in 2019 (Oğuzoğlu et al. 2010, 2012, 2019; Yeşilbağ et al. 2008, 2014; Bulut et al. 2018; Timurkan and Aydın 2019). The aim of this study was to use *5′UTR*, *N^pro^* and *E2* sequencing to examine the pestivirus species and subgenotypes circulating in samples taken from cattle with clinical findings and suspicion of BVDV infection from eastern Turkey.

## 2 Materials and Methods

### 2.1 Sample Collection and Ethics Statement

This study was approved by the Animal Research Local Ethic Committee of Van Yuzuncu Yil University (Decision No. 2022‐12‐08). A total of 110 samples were collected from 85 cattle brought to veterinary faculty animal hospitals and private animal clinics over 6 months of age with clinical suspicions of BVDV (respiratory, intestinal and genital problems) from Ağrı (*n* = 26), Iğdır (*n* = 28) and Van (*n* = 56) provinces in eastern Turkey between 2019 and 2022. These samples were stored at −80°C in the laboratory of the Department of Virology at Van Yuzuncu Yil University's Faculty of Veterinary Medicine until they were processed and analysed. It was confirmed by the animal owners that the sampled animals had not been vaccinated against BVDV.

### 2.2 Viral RNA Extraction and RT‐PCR

RNA was extracted from the samples (blood, serum, nasal/vaginal swabs and abortion materials) using commercial RNA isolation kits (EUR_X_, Poland) according to the manufacturer's instructions. The reverse transcriptase polymerase chain reaction (RT‐PCR) process was conducted using the OneStep RT‐PCR kit (Grisp, Portugal) following the manufacturer's instructions. A 288 base pair (bp) sequence for each sample of the *Pestivirus genus* was amplified by RT‐PCR using p324 and p326 primers (Table 1), as previously described (Vilcek et al. 1994). In brief, RT‐PCR was conducted with a reverse transcription step at 55°C for 15 min, followed by 35 cycles of denaturation at 95°C for 10 s, annealing at 56°C for 30 s and extension at 72°C for 30 s. Subsequently, an RT‐PCR assay for each sample was performed using BD1/BD2 (428 bp) and BD1/BD3 (738 bp) primers (Table 1) for the *N^pro^* gene region of the BVDV genome as described by Vilcek et al. (2001) with minor modifications. In addition, the complete sequence of the *E2* gene region of the BVDV‐3 genome was tested in RT‐PCR to verify the presence of this genotype in the samples using E2‐F1 and E2‐R1 primer pairs (1119 bp) under thermocycler conditions as previously described (Liu et al. 2009).

**TABLE 1:** PCR primers used to amplify viral *5′UTR*, *N^pro^* and *E2* gene sequences in this study.

Gene	Primers	Sequence (5′–3′)	Base pairs (bp)	References
*5′UTR*	P‐324	ATGCCCWTAGTAGGACTAGCA	288	Vilcek et al. (1994)
	P‐325	TCAACTCCATGTGCCATGTAC		
*N^pro^*	N^pro^‐BD1	TCTCTGCTGTACATGGCACATG	BD1‐BD2:738	Vilcek et al. (2001)
	N^pro^‐BD2	TTGTTRTGGTACARRCCGTC	BD1‐BD3:428	
	N^pro^‐BD3	CCATCTATRCACACATAAATGTGGT		
*E2*	E2‐F1	GACCTCAGTTGTAAGCCTGAG	1119	Liu et al. (2009)
	E2‐R1	CCCCCTAGCTCCTTGTTCAGTT		

### 2.3 Sequencing and Phylogenetic Analysis

The PCR‐positive products from various gene regions (*5′UTR*—288 bp, *N^pro^*—428 bp and *E2*—1119 bp) were purified using a commercial DNA purification kit (EURX, Poland) and then sequenced. Sequence analysis was carried out at BM Labosis (Ankara, Turkey) using the Sanger dideoxy sequencing method. The selected sequences were identified according to *Pestivirus* genotypes by nBLAST on the National Center for Biotechnology Information database. Subsequently, sequence analysis raw data were aligned using the Clustal algorithm in BioEdit v.7.0.5 (Hall 1999) with both each other and international sequences from GenBank. Alignments were then trimmed to a uniform length and manually edited. Poor‐quality or overly short sequences were removed, and the remaining sequences were realigned. The sequences identified in this study were compared at the amino acid level among themselves and with reference BVDV isolates from GenBank. Turkish nucleotide sequences based on *5′UTR* and *N^pro^* gene regions were used to create phylogenetic trees in MEGA version 11.0 using the maximum likelihood method and bootstrap analysis with 1000 replicates (Tamura, Stecher, and Kumar 2021). The sequence data were submitted to GenBank and received accession numbers (Table 2).

**TABLE 2:** Information on the samples, BVDV genotypes and accession numbers of the *5′UTR* and *N^pro^* gene regions for samples from cattle in eastern Turkey.

Sample ID	Sample type	Location	BVDV genotype	Accession no.
TR‐VAN‐Pest1	Blood	Van	1*a*	OR854446
TR‐VAN‐Pest2	Blood	Van	1*a*	OR854447
TR‐VAN‐Pest3	Blood	Van	1*a*	OR854448
TR‐AGR‐Pest4	Nasal swap	Ağrı	1*b*	OR854449
TR‐VAN‐Pest5	Abort material	Van	1*b*	OR854450
TR‐VAN‐Pest6	Abort material	Van	1*l*	OR854451
TR‐IGDR‐Pest7	Nasal swap	Iğdır	1*d*	OR854452
TR‐AGR‐Pesti8	Blood	Ağrı	1*a*	PP100121
TR‐AGR‐Pesti9	Blood	Ağrı	1*a*	PP100122
TR‐VAN‐BV1	Abort material	Van	1*b*	OR887473
TR‐VAN‐BV2	Abort material	Van	1*b*	OR887474
TR‐VAN‐BV3	Abort material	Van	1*b*	OR887475
TR‐AGR‐BV4	Vaginal swap	Ağrı	1*r*	OR887476
TR‐AGR‐BV5	Vaginal swap	Ağrı	1*r*	OR887477
TR‐IGDR‐BV6	Nasal swap	Iğdır	1*f*	OR887478

## 3 Results

In the RT‐PCR assay using panpesti primers (*5′UTR*—288 bp), 24 of 110 samples tested positive for pestivirus nucleic acid. The positive rates based on sample types were: 22.53% (16/71) for blood samples, 12.5% (1/8) for vaginal swabs, 26.31% (5/19) for abortion materials and 16.66% (2/12) for nasal swabs, resulting in an overall positivity rate of 21.81%. In addition, samples were tested using BVDV genome *N^pro^* gen region primers BD1/BD3 (428 bp) and BD1/BD2 (738 bp). This amplification revealed that 10 of 110 samples were positive, with positivity rates of 25% (2/8) for vaginal swabs, 15.78% (3/19) for abortion materials, 8.33% (1/12) for nasal swabs and 5.63% (4/71) for blood samples, resulting in an overall rate of 9.09%. Moreover, in this scope of the study, any positivity was not detected in the RT‐PCR using the BVDV‐3 *E2* gene region primers.

Fifteen positive amplicons (TR‐Pest1‐9 for *5′UTR* and TR‐BV1‐6 for *N^pro^*) of partial *5′UTR* and *N^pro^* coding gene regions of Turkish pestivirus from cattle were used for genotyping. After sequence analysis, the sequences of nine samples from the panpesti *5′UTR* and six samples from the *N^pro^* gene region were validated and deposited in GenBank with the corresponding accession numbers, as shown in Table 2.

**FIGURE 1:**
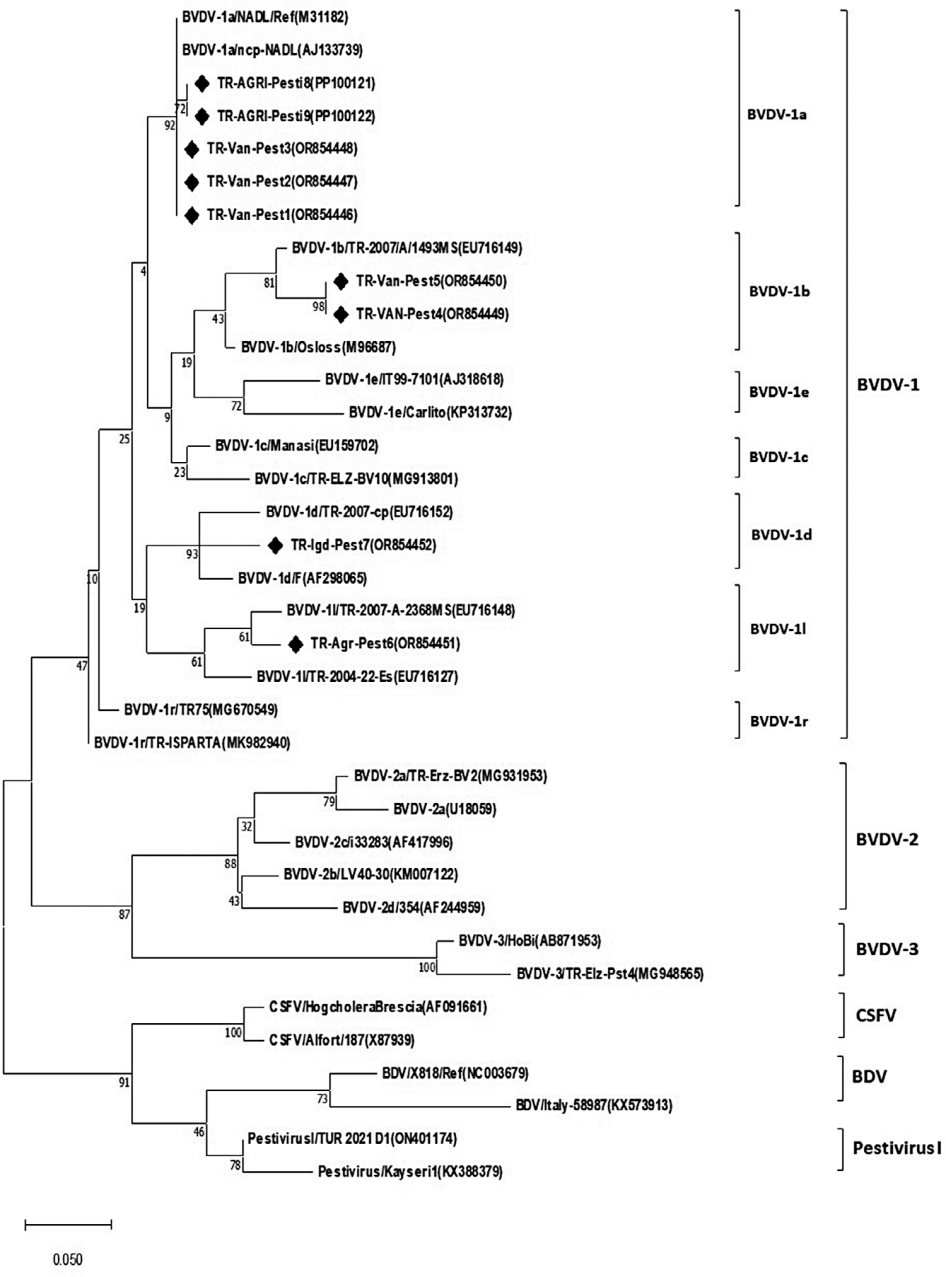
Maximum likelihood phylogenetic tree based on the *5′UTR* gene region of the pestivirus sequences (sequences found in this study are indicated by the black colour quadrilateral mark).

**FIGURE 2:**
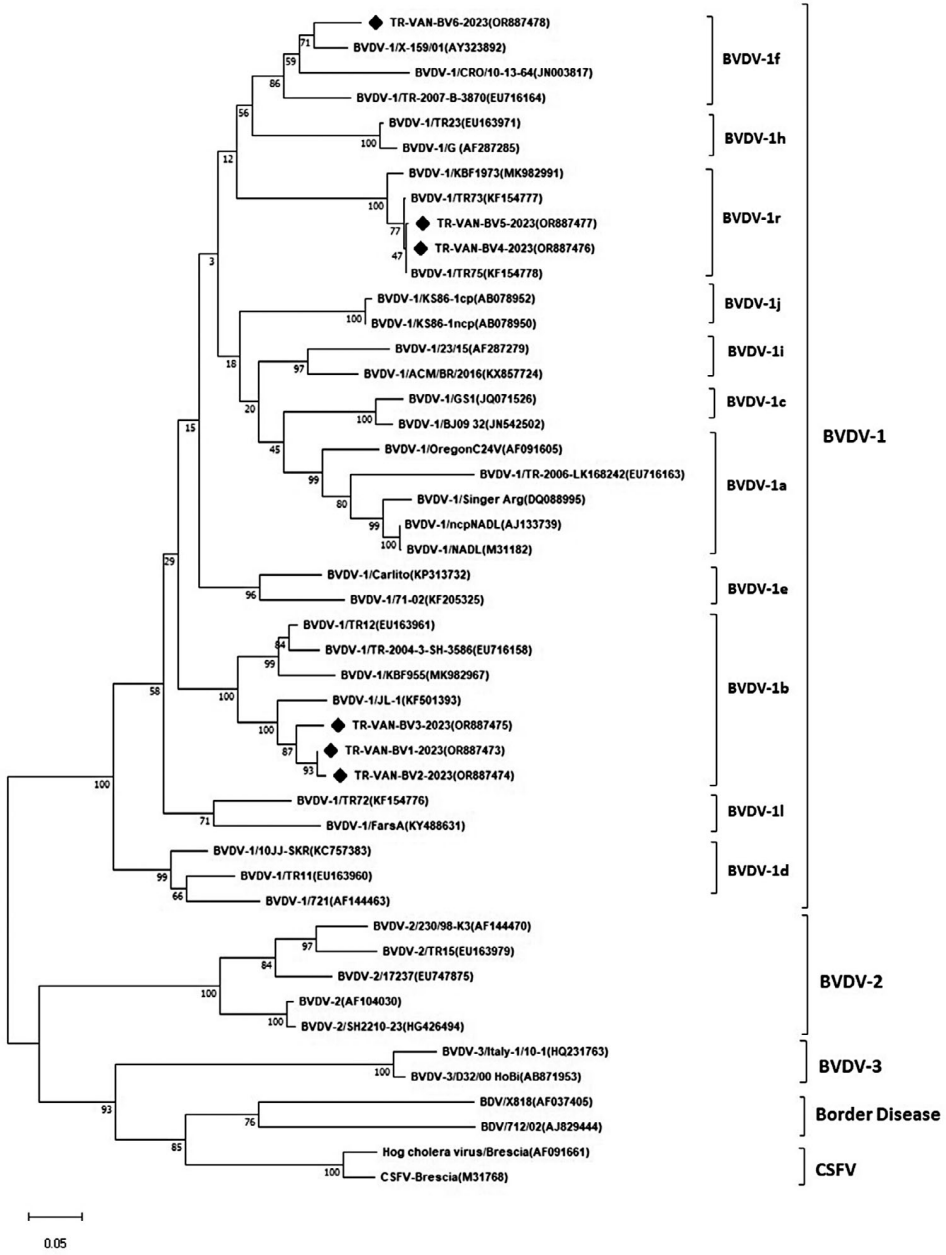
Maximum likelihood phylogenetic tree based on the *N^pro^* gene region of the pestivirus sequences (sequences found in this study are indicated by the black colour quadrilateral mark).

The investigation of the genetic divergence of BVDVs was assessed by phylogenetically analysing both the *5′UTR* and *N^pro^* sequences, which were then compared with BVDV sequences from around the world. All 15 Turkish BVDV sequences from 13 cattle in the present study were identified as BVDV‐1 (*Pestivirus A*) genotype. The phylogenetic analysis of the *5′UTR* and *N^pro^* regions showed that 33.33% (*n* = 5) of the samples were classified as BVDV‐1*a*, 33.33% (*n* = 5) as BVDV‐1*b*, 6.66% (*n* = 1) as BVDV‐1*d*, 6.66% (*n* = 1) as BVDV‐1*f*, 6.66% (*n* = 1) as BVDV‐1*l* and 13.33% (*n* = 2) as BVDV‐1*r* (Figures 1 and 2). No BVDV‐2 or BVDV‐3 genotypes were identified in this study.

## 4 Discussion

Pestivirus infection is common in the world, as well as in Turkey, with different clinical symptoms and causes significant economic losses. Studies conducted in Turkey report the presence and prevalence of pestivirus infection in different clinical findings, both serologically and virologically. Due to animal imports and animal movements in the country, BVDV sub‐genotypes identified in other countries have also been widely detected in Turkey (Yeşilbağ et al. 2008; Oğuzoğlu et al. 2010, 2012; Yilmaz et al. 2012; Yeşilbağ et al. 2014; Oğuzoğlu et al. 2019; Timurkan and Aydın 2019). Monitoring BVDV genotypes in a particular geographic area is essential both for clinical and biological reasons, given the antigenic variations among BVDV subtypes. This genetic variability is important for designing effective vaccines as well as for implementing successful infection prevention and control programs (Yeşilbağ, Alpay, and Becher 2017; Ebranati et al. 2018).

Analysis of data found that 21.81% of samples taken from animals with suspected pestivirus infection had *Pestivirus A* (BVDV‐1) infection, at least in the provinces of Ağrı, Iğdır and Van in eastern Turkey. Phylogenetic analysis of the *5′UTR* and *N^pro^* regions (Figures 1 and 2) revealed that all 15 Turkish isolates from BVDV‐positive samples belonged to the BVDV‐1 genotype. These isolates were further classified into subgroups 1*a*, 1*b*, 1*d*, 1*f*, 1*l* and 1*r*, despite originating from different provinces. It also found that BVDV‐1*a*, 1*b* and 1*r* subgenotypes are prevalent in the region analysed, and among these, BVDV‐1*a* (33.33%) and BVDV‐1*b* (33.33%) are more dominant in the respective regions. The nine Turkish sequences (*5′UTR*) showed a homology rate of 80.1%–99% among themselves, while the six sequences (*N^pro^*) had a genomic nucleotide identity of 87.9%–96.1%. Based on the *5′UTR* gene region, the subgenotypes of nine isolates were identified as BVDV‐1*a* (*n* = 5), 1*b* (*n* = 2), 1*d* (*n* = 1) and 1*l* (*n* = 1). In contrast, analysis of the *N^pro^* gene region identified six isolates as BVDV‐1*b* (*n* = 3), 1*r* (*n* = 2) and 1*f* (*n* = 1). This finding shows that it is partially compatible with studies that found BVDV‐1 subgroups in Turkey.

The *5′UTR* is commonly used for BVDV genotyping due to its highly conserved regions, which aid in amplifying various BVDV strains (Vilcek et al. 2004, Vilcek, Durkovic, et al. 2005). Considering the high mutation rate of BVDV and the recent emergence of *Pestivirus H*, also known as the HoBi‐like virus, utilising this region in genotyping studies is particularly crucial. Nevertheless, its utility for genotyping has been questioned (Becher et al. 2003). This highlights that BVDV variability can arise from mutation and recombination, causing clustering differences depending on the region analysed.

Phylogenetic analysis of pestiviruses is essential for classifying new viruses and uncovering their evolutionary history. Studies on the genetic characterisation of pestiviruses in Turkey reveal that the BVDV‐1 genotype, particularly subtype BVDV‐1*l*, is dominant (Yeşilbağ et al. 2008; Oğuzoğlu et al. 2012; Timurkan and Aydın 2019). Globally, BVDV‐1*b* is the most common subtype, followed by 1*a* and 1*c* (Yeşilbağ, Alpay, and Becher 2017; Zhou et al. 2022). In Turkey, a range of BVDV subtypes have been detected, including BVDV‐1*a*, 1*b*, 1*c*, 1*d*, 1*f*, 1*h*, 1*i*, 1*l*, 1*r*, 1*v*, BVDV‐2*a*, 2*b* and recently detected BVDV‐3. This study confirms previous findings on dominance of the *Pestivirus A* (BVDV‐1), identifying BVDV‐1*a* (33.33%, *n* = 5), BVDV‐1*b* (33.33%, *n* = 5) as the most common subtypes in the mentioned provinces in eastern Turkey, followed by 1*r* (*n* = 2), 1*d* (*n* = 1), 1*f* (*n* = 1) and 1*l* (*n* = 1). Comparing all those isolates with other studies, such as BVDV‐1*a*, 1*b*, 1*d*, 1*f*, 1*l* and 1*r* subtypes, they appear to be compatible among each other (Yeşilbağ et al. 2008; Oğuzoğlu et al. 2010; Oğuzoğlu et al. 2012; Yilmaz et al. 2012; Sarikaya et al. 2012; Oğuzoğlu et al. 2019; Timurkan and Aydin 2019).

BVDV infection continues to significantly impact the cattle industry worldwide. Control measures primarily involve herd screening, removal of PI animals and vaccination of female cattle for fetal protection. In Turkey, where vaccines primarily target BVDV‐1*a* and 1*b* strains, recent data suggest that an eradication program tailored to the local BVDV‐1*l* and 1*f* subgenotypes is overdue. The study highlights the challenge posed by BVDV's genetic diversity, which may lead to vaccine failure and stresses the importance of developing vaccines effective against local strains. The detection of European BVDV‐1 subgroups in Turkey, likely from cattle imports, underscores the need for vaccines suited to the region. Ongoing study is evaluating vaccine efficacy and conducting cross‐neutralisation studies, with a focus on expanding molecular studies to better support eradication efforts. In addition, the study emphasises the importance of further BVDV characterisation in Asia, the Middle East and neighbouring countries to enhance the understanding of global pestivirus epidemiology.

## 5 Conclusion

In summary, this study investigated the genotypes and variability of BVDVs in cattle from eastern Turkey. Identifying *Pestivirus A* (BVDV‐1) with several subgenotypes points to an increase in BVDV‐1 genetic diversity. The findings underscore the importance of screening for BVDV, given its role in causing intestinal, genital and respiratory diseases, along with significant economic losses. The study emphasises the need for effective control and eradication strategies, including vaccination, to manage the increasing genetic diversity of BVDV.

## Author Contributions

**Fatima Abounaaja**: conceptualisation, investigation, visualisation, formal analysis, software and writing–review. **Ali R. Babaoglu**: conceptualisation, investigation, writing–original draft, supervision, validation, visualisation, writing–review and editing, software, project administration.

## Data Availability

The data that support the findings of this study are available from the corresponding author upon reasonable request.
